# Harnessing the cDC1-NK Cross-Talk in the Tumor Microenvironment to Battle Cancer

**DOI:** 10.3389/fimmu.2020.631713

**Published:** 2021-02-19

**Authors:** Johanna Bödder, Tasmin Zahan, Rianne van Slooten, Gerty Schreibelt, I. Jolanda M. de Vries, Georgina Flórez-Grau

**Affiliations:** Department of Tumor Immunology, Radboud Institute for Molecular Life Sciences, Radboud University Medical Center, Nijmegen, Netherlands

**Keywords:** natural killer cells, conventional type 1 DCs, cross-talk, tumor microenvironment, immunotherapy

## Abstract

Immunotherapeutic approaches have revolutionized the treatment of several diseases such as cancer. The main goal of immunotherapy for cancer is to modulate the anti-tumor immune responses by favoring the recognition and destruction of tumor cells. Recently, a better understanding of the suppressive effect of the tumor microenvironment (TME) on immune cells, indicates that restoring the suppressive effect of the TME is crucial for an efficient immunotherapy. Natural killer (NK) cells and dendritic cells (DCs) are cell types that are currently administered to cancer patients. NK cells are used because of their ability to kill tumor cells directly *via* cytotoxic granzymes. DCs are employed to enhance anti-tumor T cell responses based on their ability to present antigens and induce tumor-antigen specific CD8^+^ T cell responses. In preclinical models, a particular DC subset, conventional type 1 DCs (cDC1s) is shown to be specialized in cross-presenting extracellular antigens to CD8^+^ T cells. This feature makes them a promising DC subset for cancer treatment. Within the TME, cDC1s show a bidirectional cross-talk with NK cells, resulting in a higher cDC1 recruitment, differentiation, and maturation as well as activation and stimulation of NK cells. Consequently, the presence of cDC1s and NK cells within the TME might be of utmost importance for the success of immunotherapy. In this review, we discuss the function of cDC1s and NK cells, their bidirectional cross-talk and potential strategies that could improve cancer immunotherapy.

## Introduction

Cancer immunotherapy is an approach that aims to activate the immune system to fight cancer. The immune system consists of many different cell types interacting with each other. Natural Killer (NK) cells and dendritic cells (DCs) are two cell types used for immunotherapy that are currently being tested in the clinic.

NK cells are granular innate lymphoid cells that display rapid, contact-dependent cytotoxic activities against viral-infected and cancer cells without prior sensitization. In general, NK cells rapidly accumulate at sites of inflammation, where they recruit other immune cells *via* cytokine and chemokine secretion. After activation, NK cells induce lysis or apoptosis in mutated cells by releasing granules containing cytotoxic granzymes. Activation occurs in an antigen-independent manner that is regulated by a tight balance of activating and inhibitory germline-encoded surface receptor ligation (1). Activating receptors bind to ligands (e.g., CD155, CD112) upregulated on tumor cells. Inhibitory receptors recognize major histocompatibility complex (MHC) class I, which is expressed by all nucleated cells, and upon binding, suppress NK cell activation. Hence, the highly diverse receptor repertoire on NK cells and the balance of activating and inhibitory receptors determine the magnitude of NK cell-mediated cytotoxicity and allow them to remain tolerant towards healthy cells (1–3). DCs, are a heterogeneous cell population which main function is to initiate an immune response. Immature DCs act as sentinels as they take up antigens, undergo a maturation process, and present these antigens on MHC molecules to naive T cells in lymph nodes. In general, antigen presenting cells present endogenous antigens on MHC class I (MHC-I), and exogenous antigens on MHC class II molecules (MHC-II) and thereby prime and activate CD4^+^ and CD8^+^ T cells, respectively (4). However, DCs have the unique capacity to present exogenous antigens on MHC-I molecules to CD8^+^ T cells, a process known as antigen cross-presentation.

NK cells are exploited as immunotherapeutic tool due to their cytotoxic and immunomodulatory functions and DCs because they are able to antigen-specifically activate T cells. However, both NK cell and DC functions can be restricted by the immunosuppressive tumor microenvironment (TME). In this review, we describe the main features of NK cells and a very rare type of DC, cDC1s, and emphasize the importance of these cell types within the TME. We focus on how to exploit cDC1s and NK cells and their interaction as a potential target to enhance efficacy of cancer immunotherapy.

## Dendritic Cells

**Figure 1:**
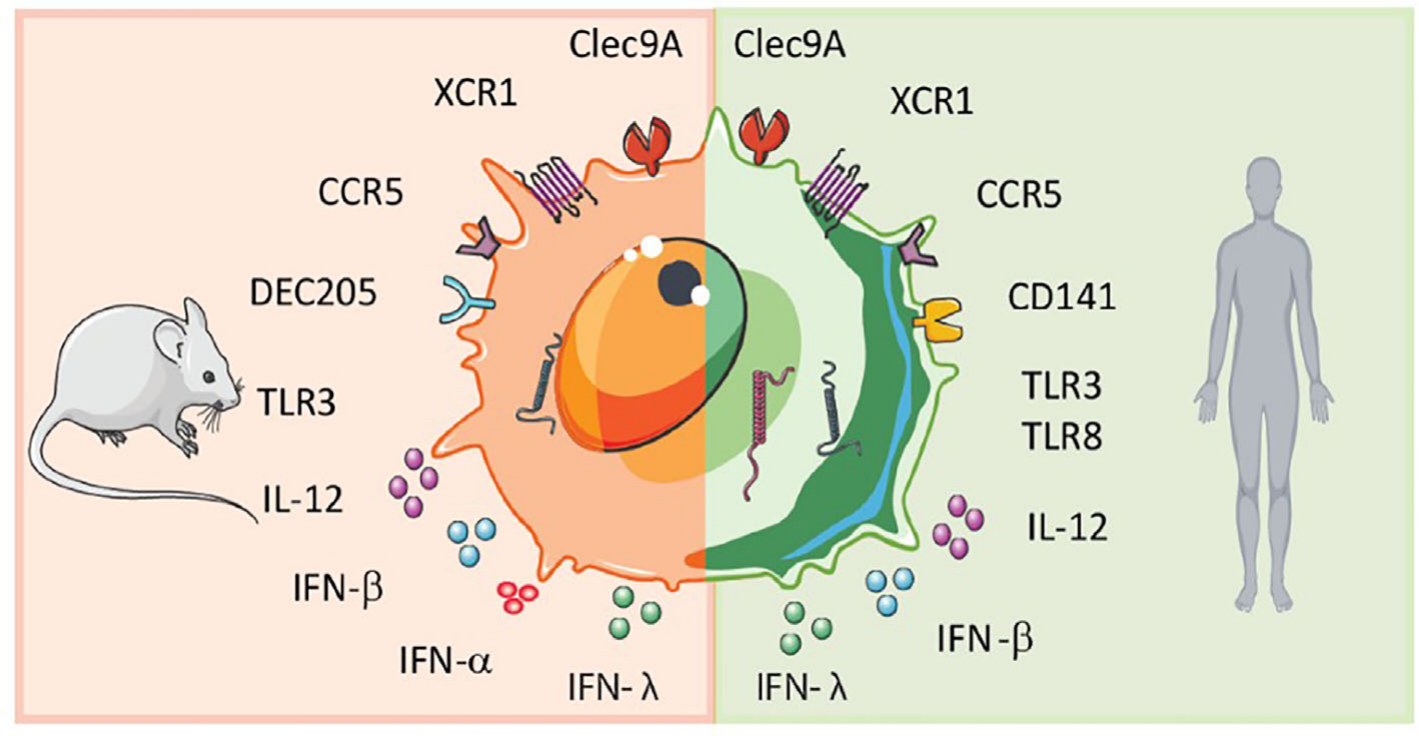
Scheme of murine and human cDC1 features. Human and mouse cDC1s display similarities but also differences in surface receptor expression and cytokine secretion.

Both in humans and mice, circulating blood DCs have been classically divided into myeloid or conventional DCs (cDCs), and plasmacytoid DCs (pDCs). The DC subsets are classified by surface marker expression and different functional properties. Human pDCs express CD123, CD303 (BDCA-2) and CD304 (BDCA-4) as distinctive markers and are known for the production of large amounts of type 1 interferon (IFN-I) especially important for strong anti-viral responses (5). Conventional DCs express the common myeloid markers: CD11c, CD11b, CD33, and CD13 and are efficient in antigen presentation and T cell activation (5). They can be subdivided into type 1 conventional DCs (cDC1s) and type 2 cDCs (cDC2s) (6, 7). In humans, cDC1s express CD141 (BDCA-3) and cDC2s express CD1c (BDCA-1) (5). In mice, cDC2s are CD11b^+^, and cDC1s are characterized by CD8α^+^ or CD103^+^ expression (8). Genome-wide association studies of human and mouse cDC1s revealed phenotypic similarities, including expression of nectin-like protein 2 (Necl2), C-type lectin CLEC9a, and the XC chemokine receptor 1 (XCR1) as well as toll-like-receptor 3 (TLR-3) (**Figure 1** ) (9–14). Hence, human cDC1s are considered to be the equivalent of the mouse CD8α^+^ D C subset (10–13, 15, 16) (**Figure 1**).

### cDC1s Phenotypic Characterization

Whereas in mice, cDC1s are the most abundant DC subset, human cDC1s are the rarest with approximately 0.03% of PBMCs and lymphoid and non-lymphoid cells are cDC1s (10, 11). This low occurrence of cDC1s resulted in difficulties to characterize them phenotypically. Initially, CD141 (BDCA-3) was described as a distinctive marker for cDC1s. However, CD141 was also found on cDC2s and other myeloid cells like monocytes (17). Therefore, many research groups sought to redefine subset division and attempted to identify conserved and exclusive markers across species. In this context, single-cell RNA-sequencing data recently demonstrated that cDC1s indeed form a single, separate cluster with CLEC9a as a specific marker for human cDC1s (18). CLEC9a is a receptor for necrotic cell-derived antigens (19). Yet, also CLEC9a is not exclusively expressed on cDC1s (13, 20, 21). The chemokine receptor XCR1 was established to be a specific marker for cDC1s. It is exclusively expressed on both human and mouse cDC1s (12, 13, 22–24). XCR1 mediates chemotaxis of cDC1s towards CD8^+^ T cells and NK cells, because they are the main producers of the ligand of XCR1, XC chemokine ligand 1 (XCL1), the ligand of XCR1 (12, 23). Besides exclusive expression of XCR1, cDC1s have a higher expression of the TLR-3 compared to other DCs subsets. TLR-3 signaling triggers IRF3/7, leading to IFN-ß secretion, thereby providing cDC1s with the enhanced capability to initiate T helper 1 responses (10, 11). As a result of the expression of CLEC9a and XCR1, cDC1s can take up necrotic cell-derived antigens and can migrate towards CD8^+^ T cells.

### cDC1s Activate T Cells *via* Cross-Presentation

Cross-presentation of extracellular antigens is essential to activate CD8^+^ T cells specific for antigens derived from tumor cells (25). In mice, cDC1s are the only subset that cross-present antigens. Depletion of cDC1s, for example using *Batf3* knockouts, results in loss of cross-presentation. Mice lacking cDC1s have less tumor-specific CD8^+^ T cells hence an impaired anti-tumor response (24, 26–29). Of note, depletion of cDC1s leads, besides to diminished cross-presentation also to loss of other functions mediated by cDC1s like attracting CD8^+^ T cells *via* XCL1-XCR1 interactions as well as less IL-12 and IFN-β since it is produced by cDC1s upon TLR-3 triggering. These processes also enhance anti-tumor responses and are less efficient upon depletion of cDC1s (6, 24).

In line with data obtained from mouse experiments, human cDC1s are shown to be highly efficient at antigen cross-presentation (10–13). However, in humans, cDC1s are not the only subset capable to cross-present. Plasmacytoid DCs, cDC2s and monocyte-derived DCs are also able to cross-present (30–34). However, data show that cDC1s are the most competent DC subset to cross-present antigens, especially of necrotic cells after uptake *via* CLEC9A (27, 35). The efficiency of cross-presentation of cDC1 is enhanced after triggering of TLR3 (11). Hence, human cDC1s are also highly specialized to cross-present antigens from necrotic tumor cells and to initiate a tumor antigen specific CD8^+^ T cell response (36, 37).

## Natural Killer Cells

Human blood NK cells are a heterogeneous immune cell population classified by surface receptor expression of CD56 into two main functionally different subsets. CD56^high^ NK cells secrete high levels of IFN-γ and other cytokines important for immunoregulatory functions, but produce low levels of perforin (38). Furthermore, CD56^high^ NK cells express high levels of natural killer group 2A (NKG2A) and C-C chemokine receptor type 7 (CCR7) and low levels of Fcγ receptor IIIa (CD16) and killer cell immunoglobulin-like receptors (KIRs) (38, 39). In contrast, CD56^low^ NK cells display a high expression of KIR and CD16 receptor. The receptor CD16 enables CD56^low^ NK cells to mediate antibody-dependent cellular cytotoxicity (ADCCs). This CD16 expression together with the secretion of perforin and granzyme B provides CD56^low^ NK cells with efficient killing abilities (38).

**Figure 2:**
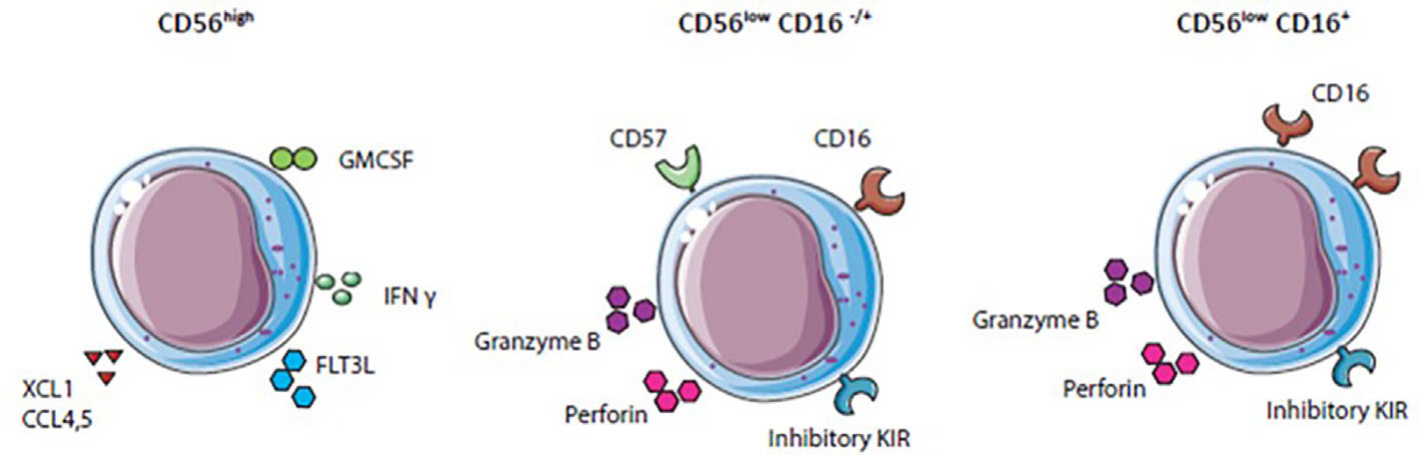
Natural killer (NK) cell subset differentation: NK cell subsets express variouus activating and inhibitory receptors and secrete different cytokines.

In general, NK cells display either killer (CD56lowNK cells) or helper (CD56high) functions against virus-infected or tumor cells due to activating and inhibitory receptor signaling and cytokine secretion (**Figure 2**). Inhibitory KIR and NKG2A receptors recognize MHC-I molecules. In the case of absent, changed, or mismatched MHC-I expression, the inhibitory receptor signal is lost, and NK cells get activated. The diversity of inhibitory receptors expressed on NK cells allows to recognize the high polymorphism of the *MHC class I* genes resulting in NK cell tolerance for healthy cells (3). Virus-infected or tumor cells often show gradually or complete loss of MHC-I expression to escape cytotoxic T cell recognition, simultaneously leading to a lack of inhibitory signal and NK cell activation (38, 40). Besides NK cell activation by loss of inhibitory signals, ligands overexpressed on mutated cells engaging with activating receptors such as natural cytotoxicity receptors (NKp30, NKp44, and NKp46) and NKG2D also lead to NK cell activation (41). Exposure of NK cells to cytokines such as IL-2 or IL-15 enhances activating receptor upregulation and promotes survival and proliferation (42–44). Activated NK cells can kill by forming a synapse with target cells and releasing perforin and cytotoxic granules. Perforin penetrates the membrane of target cells and granules containing cytotoxic granzymes enter target cells to provoke programmed cell death (45, 46). Another killing mechanism of NK cells is *via* ADCC. CD16 on NK cells recognize IgG antibody-coated tumor cells upon which granules are released by NK cells and target cells are killed (47). Some anti-tumor therapies based on neutralizing antibodies such as Rituximab or Trastuzumab demonstrate that the clinical benefit is partly mediated by ADCC (47).

Immunomodulatory functions, mainly ascribed to CD56^high^ NK cells are the secretion of different cytokines, chemokines, and growth factors, for example, IFN-γ, TNF-α, fms-like tyrosine kinase 3 ligand (FLT3L), Chemokine (C-C motif) ligand 3 (CCL3), CCL4, CCL5, granulocyte-macrophage colony-stimulating factor (GM-CSF) and XCL1 (41). These factors attract other immune cells like DCs, induce Th1 polarization, and CTL responses against target cells (41, 48). NK cells are essential for anti-tumor immune responses especially for eradication of MHC-I negative tumors. Different strategies to boost NK cell activity, particularly in the suppressive TME, are currently investigated.

## Tumor Microenvironment and Immune Escape Mechanisms

Escaping immune surveillance is one of the key hallmarks of cancer (49). Different suppressive mechanisms are employed by cancer cells to bypass immune system attacks. These mechanisms include the upregulation of co-inhibitory ligands (such as programmed death-ligand 1, PD-L1), down-regulation or loss of MHC-I, or secretion of immunosuppressive factors like IL-10, VEGF, and TGF-β (50–54).

The composition of the TME differs between cancer type and patient and plays an essential role in the tumor immune escape. This highly heterogeneous TME is composed of tumor cells, blood vessels, tumor-infiltrating lymphocytes, and other immune cells, fibroblasts, endothelial cells, signaling molecules (cytokines, chemokines), and the extracellular matrix (55). Tumors can be divided into “hot” and “cold” depending on the presence or absence of effector immune cells within the tumor. The occurrence of effector immune cells, for example T cells which directly can eliminate tumor cells, impacts patient outcome (55). In some cancer types, such as epithelial ovarian cancer and colorectal cancer T cell infiltration, specifically in the tumor has been associated with positive clinical effects (56–58). Moreover, pre-existing CD8^+^ T cells within the tumor are crucial for tumor regression upon PD-1 checkpoint inhibition (59). In contrast to CD8^+^ T cells, regulatory T cells, myeloid-derived-suppressor cells, or tumor-associated macrophages inhibit the immune response and are therefore associated with tumor growth and a poor clinical outcome (60, 61).

### NK Cells and DC Within the TME

Next to the presence of CD8^+^ T cells, the occurrence and active state of NK cells in the tumor are related to a positive prognostic outcome (62–64). In some types of cancer such as head and neck or prostate cancer, a high ratio of tumor infiltrating NK cells is associated with a positive clinical outcome for patients, whereas in non-small-cell lung cancer, the presence of NK cells within the TME has no clinical impact due to down-regulation of activating receptors on the infiltrated NK cells (63, 65). NK cells isolated from prostate cancer are mainly CD56^high^ and display reduced cytotoxicity because of an increased inhibitory and decreased activating receptor expression pattern. In non- small cell lung carcinoma CTLA-4 expression is upregulated on NK cells in the TME compared with healthy tissue NK cells and negatively effects DC maturation (66). This unfavorable activating and inhibitory receptor expression pattern have been implicated as cause for impaired killing activity of NK cells in the TME (67, 68).

NK cells isolated from tumors differ phenotypically and transcriptomically from NK cells isolated from blood (69). These variations can arise from several cellular and soluble factors present within the TME, which influence phenotype, functionality, and migration characteristics of infiltrated NK cells (63, 66, 70). Soluble factors influencing NK cell cytotoxicity and infiltration into the TME for instance TGF-β and Prostaglandin E2 (PGE2) are secreted by immunosuppressive cells such as regulatory T cells, tumor-associated macrophages, myeloid-derived-suppressor cells (68, 71–73). TGF-β suppresses NK cell cytotoxicity by inhibiting IFN-γ secretion, down-regulating activating receptor expression, and the adapter molecule DAP12 (74–76). PGE2 hampers NK cell cytotoxicity by decreasing IFN-γ secretion, thereby facilitating cancer progression (77, 78). Other factors within the TME, like hypoxia, high expression of checkpoint receptor ligands, and chronic activation of activating receptors, can cause functional exhaustion of NK cells (79–81). Exhausted NK cells show a dampened inflammatory cytokine secretion pattern, a reduced activity due to decreased activating receptor expression and an upregulation of inhibitory receptors and checkpoint receptors such as PD1, TIM3, CD69, and LAG3 (63, 68, 82, 83).

**Figure 3:**
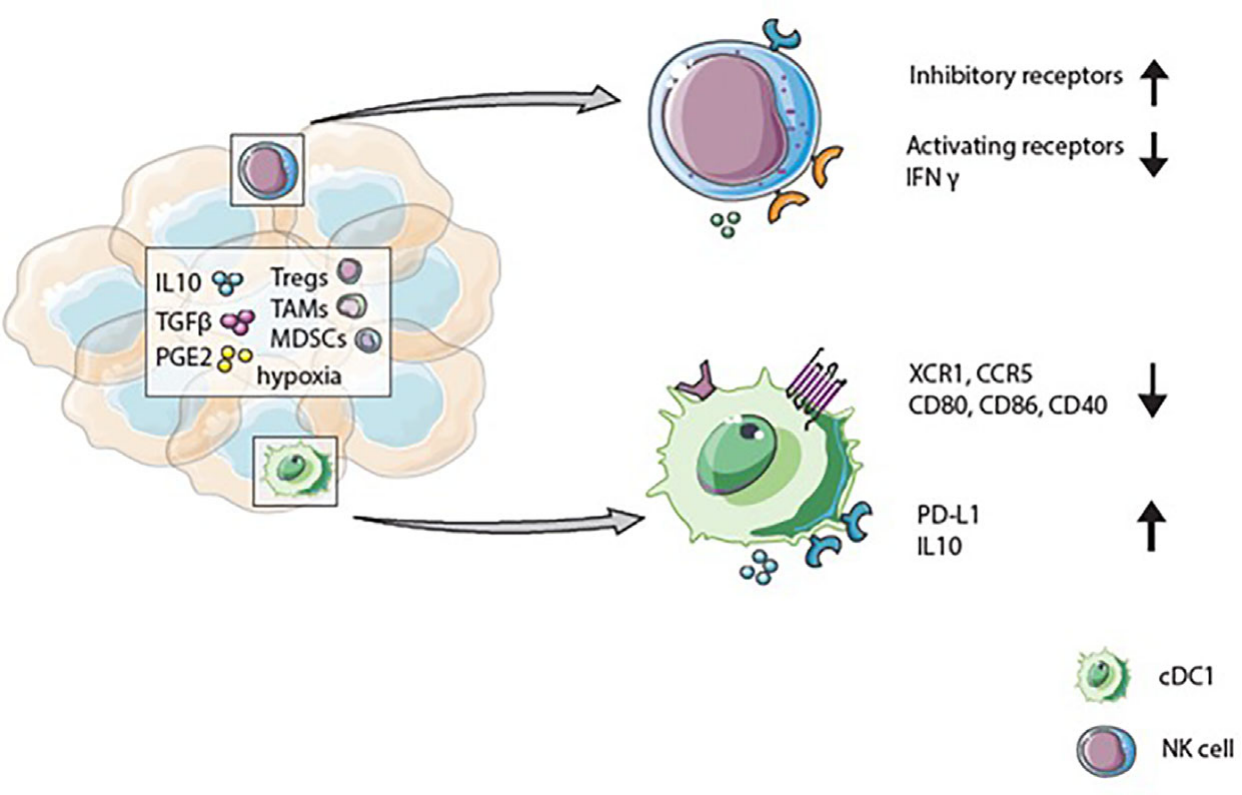
The tumor microenvironment influences cDC1 and natural killer (NK) cell phenotype and function. Immunosupressive factors like IL-10, TGF-β, and PGE2 can be secreted by tumor- and immune cells such as regulatory T cells (Tregs), tumor-associated macrophages (TAMs), or myeloid-derived-suppressor cells (MDSCs) present in the tumor microenvironment (TME). These factors, and hypoxia, can upregulate inhibitory receptors and decrease activating receptors on NK cells. Together with diminished IFN-γ secretion, the changed receptor expression results in reduced cytotoxicity of NK cells. Due to immunosuppressive factors in the TME cDC1s express low levels of XCR1 and CCR5 and display an immature phenotype with reduced CD80, CD86, and CD40 expression. Whereas checkpoint receptors and anti-inflammatory cytokine expression are upregulated.

Immunosuppressive factors within the TME also strongly influence the function of DCs within this microenvironment (**Figure 3**). Tumor-infiltrated DCs are less efficient in antigen presentation and cytokine production upon TLR stimulation than peripheral blood DCs (84, 85). Factors in the TME, including IL-10, IL-6, TGF-ß, and PGE2, modulate DCs, for example, inhibition of DC maturation. Hence, tumor-infiltrated DCs often display an immature phenotype with a low expression of co-stimulatory markers: CD80, CD86, and CD40, while co-inhibitory markers like PD-L1 and TIM3 are upregulated. Consequently, DCs within the TME are often associated with immunosuppressive and impaired functions (86–88). Notably, the infiltration of pDCs is associated with poor prognosis in breast- and ovarian cancer (89, 90). In contrast, cDC1s in the TME have been linked with favorable patient survival (91). Several reports indicate that cDC1s in tumors are associated with a higher number of CD8^+^ T cells within the TME, better prognosis of cancer patients, and immunotherapeutic success (36, 92–95). In addition to the presence of CD8^+^ T cells, the occurrence of cDC1s in the TME also correlates with high numbers of NK cells and better overall survival of melanoma patients (14, 94, 96). Positive effects of cDC1s within the TME seem to be partly mediated *via* their IL-12 secretion which is essential for the induction of T-helper 1 (Th1) responses and CD8^+^ T cell activation, both of which are crucial for a long-lasting anti-tumor response (97). Moreover, IL-12 effects IFN-γ production by NK cells. The lack of IL-12 secreting cDC1s in mice leads to reduced IFN-γ production by NK cells and growth of metastases (98). In human breast cancer, IL-12 expression correlates with cDC1s and cytotoxic effector molecules like *IFNG* (99). Nevertheless, positive effects of the presence of cDC1s can be negatively affected by different factors in the TME. For example, in mice, tumor-infiltrating cDC1s show increased PD-L1 expression upon antigen uptake and IFN-γ stimulation. High PD-L1 expression protects cDC1s from killing by CD8^+^ T cells. However, within the TME, this protective high PD-L1 expression on cDC1s can lead to a diminished anti-tumor immune response (100). Macrophage-derived IL-10 inhibits IL-12 production by cDC1s during chemotherapy, resulting in a lower CD8^+^ T cell cytotoxicity and, tumor progression in a mammary carcinoma mice model (99). A factor that affects the presence of cDC1s within the tumor is cyclooxygenases (COX), an enzyme crucial for PGE2 production. In mice, increased COX levels lead to diminished cDC1 numbers within melanoma tumors and a dampened activity by suppressed IL-12 production. COX inhibition, together with anti-PD-1 treatment, enhanced eradication of tumors. Changed immune cell infiltration due to COX expression is further confirmed in various tumor types in mice and in human tumors; COX levels correlate negatively with immune cell infiltration (101). Even though, cDC1s in the TME positively affect patient outcome different factors in the TME suppress their functional properties.

### Migration Patterns of NK Cells and cDC1s

The migration of immune cells towards lymph nodes and solid tumors is essential for proper immune activation and cancer cell elimination (55, 102). In general, the attraction and migration of immune cells occur through chemokine production and the interaction with their receptors (55). DC migration to lymph nodes is mediated by CCL19 and CCL21, the ligands for CCR7, where they present antigens to T cells and induce an antigen-specific immune response (103). The expression of CCR7 on cDC1s is crucial for migration and therefore important for antigen trafficking to lymph nodes. The lack of CCR7 expression in mice results in reduced numbers of cDC1s in the draining lymph node and increased tumor growth compared to wild type mice. In melanoma patients, cDC1s in the TME express high levels of CCR7, which predict T cell infiltration and improves clinical outcome, underscoring the importance of CCR7 expressing cDC1s for antigen trafficking and T cell priming (104).

After antigen specific activation of T cells in the lymph nodes these effector cells should migrate to the tumor to be able to kill tumor cells. Differences in chemokine and cytokine expression between hot and cold tumors impact the infiltration of effector cells (55, 105, 106). Hot tumors express high gene- and protein levels of chemokines CXC ligand (CXCL)9, CXCL10, and CXCL11 (106). All these three chemokines are secreted by DCs and bind to CXCR3 expressed on activated T- and NK cells. The high presence of CXCL9, CXCL10, and CXCL11 in the TME is associated with favorable clinical outcome in some cancers in humans (107). Interestingly, cDC1s are the primary source of those chemokines, underscoring the importance of cDC1s to attract effector cells towards the TME (95). For T cell chemotaxis into the tumor, the CXCR3/CXCL9,10,11 axis seems crucial (108, 109). For NK cells the role of CXCR3 for tumor infiltration is less straight forward. CXCR3 expression differs per NK cell subset, which influences their migration pattern. CD56^high^ NK cells express CCR7 and CXCR3, CD56^low^ NK cells express CXCR1 and CXCR2 (107, 110, 111). In breast and lung cancer, CXCL9 and CXCL10 mediate directed migration of CD56^high^ NK cells, but not of cytotoxic CD56^low^ NK cells (112). Moreover, suppressive factors present in the TME can influence chemokine production leading to altered NK cell migration. TGF-β favors CD56^high^ and dampens CD56^low^ NK cell recruitment. TGF-β decreases chemokine secretion of CXCL2, CX_3_CL1, and CXCL1 which attract CD56^low^ and increases chemokine production, which recruit CD56^high^ (CXCL9, CXCL10, CXCL11, and CCL5) (107, 110, 113). Even though factors in the TME can influence migration patterns of effector cells, the secretion of chemokines by cDC1s seem to be crucial for the recruitment of T cells and NK cells.

## Cross-Talk cDC1s and NK Cells

The cross-talk between NK cells and cDC1s is bidirectional and NK cells as well as T cells can recruit cDC1s into the tumor *via* chemokine secretion and thereby promote anti-tumor immunity (14). In both preclinical and clinical settings, cDC1s are found enriched in TMEs with a specific chemokine profile. This profile includes XCL1, mainly secreted by tumor resident CD56^low^ NK cells, and CCL4 and CCL5 mostly produced by CD56^low^ and CD56^high^ NK cells and CD8^+^ T cells (14). cDC1s express the receptors for those chemokines, namely XCR1 and CCR5 (14, 23). Indicating the importance of NK and CD8^+^ T cells for intratumoral migration of cDC1s due to XCL1, CCL5, and CCL4 (14). NK cells stimulated with IL-18 and IFN-α attract immature DC based on CCR5 expression, and stimulate DCs to increase CXCL9, CXCL10, and CCL5 production, promoting the attraction of effectors cells (114). *In vitro*, XCR1 and CCR5 expression on cDC1s is downregulated by PGE2, diminishing responsiveness to XCL1 and CCL5. In addition, PGE2 inhibits XCL1 and CCL5 secretion by NK cells, underlining the role of PGE2 as an immunosuppressive mediator to interfere with cDC1s migration to the tumor (14). In human tumors, cDC1 and NK cell gene signatures correlate with CCL5 and XCL1 gene expression and with CD8^+^ T cell infiltration. Further, NK cell and cDC1 gene signatures in the TME correlate positively with patient survival (14).

**Figure 4:**
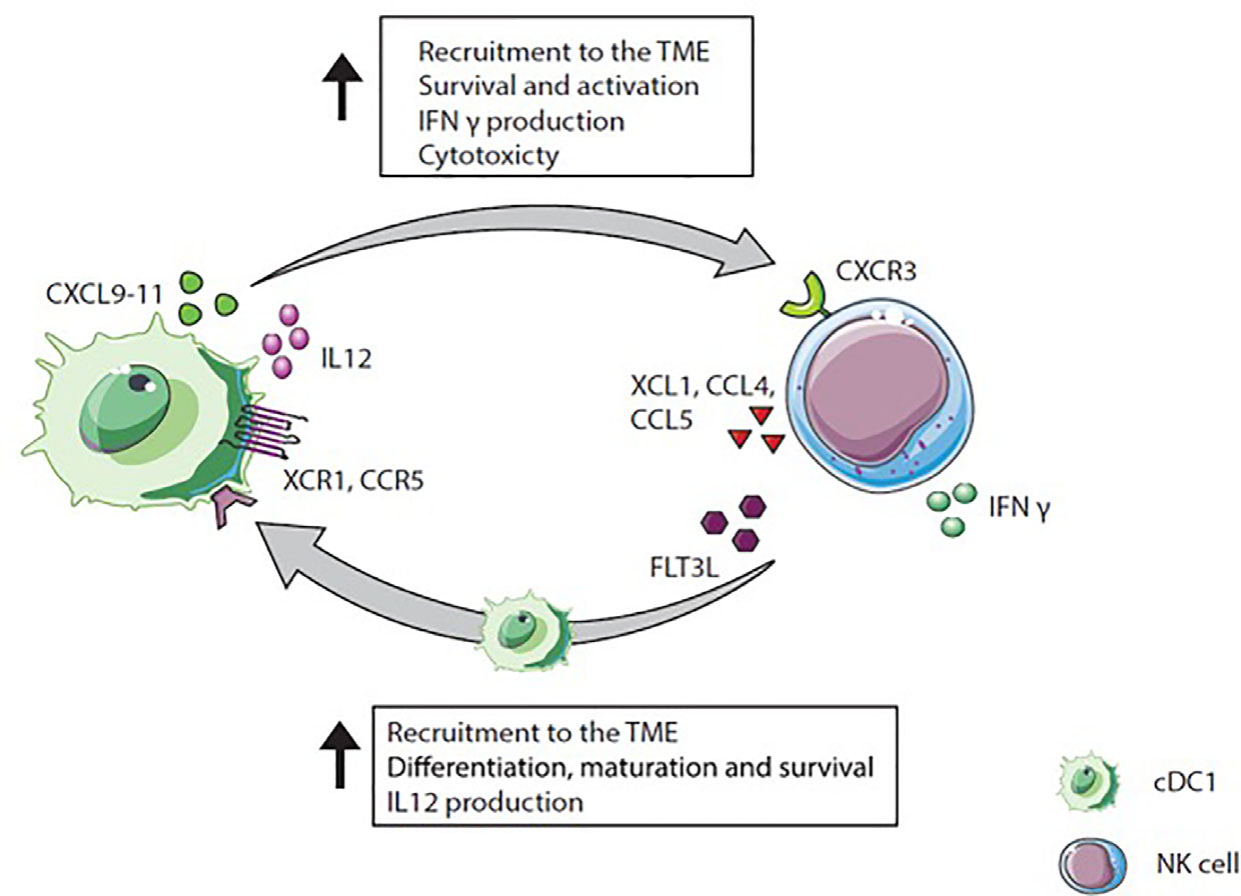
Natural killer (NK) cell and cDC1 cross-talk. NK cells secrete XCL1, CCL4, and CCL5 which attract XCR1 and CCR5 expressing cDC1s. Moreover, NK cells produce FLT3L, a differentiation factor for precursor DCs to cDC1s recruit NK cells *via* CXCL9-11 secretion into the TME. In a positive feedback loop, IL-12 produced by cDC1s activates IFN-γ extraction by NK cells, which again increases IL-12 secretion by cDC1s.

The cross-talk between NK cells and cDC1s influences the migration pattern of both cell types in addition to multiple other mechanisms by which DCs and NK cells interact (**Figure 4**). One mutual process is the maturation of DCs initiated by NK cells. Mature DCs release cytokines (IL-2, IL-12, or IL-18), which provoke NK cells to produce IFN-γ, TNF-α, or GM-CSF. These cytokines promote DC maturation (115, 116). Besides cytokine secretion, NK cells mature DCs *via* the ligation of CD40/CD40L (117). Upon CD40/CD40L ligation, the membrane bound IL-15 expression on DCs is upregulated promoting proliferation of NK cells (118, 119). The expression of CD40L on NK cells is regulated by IL-12 and IFN-γ and might determine cytotoxicity of NK cells (117, 120). Indeed, in a mouse tumor model, IL-12 and IFN-γ inhibition resulted in the down-regulation of CD40L expression on NK cells and diminished NK cell cytotoxicity (120).

Besides maturation factors, NK cells produce the differentiation factor FLT3L, which stimulates cDC1 survival, differentiation, and recruitment and thereby positively influence cDC1s in the TME (94, 121). Interestingly, intratumoral NK cells in humans and mice are a major source of FLT3L, which maintains DC viability within the TME (94). The depletion of NK cells in mice reduced the frequency of cDC1s within the tumor, indicating that FLT3L production by NK cells is required for stable cDC1 numbers within the TME. In humans, genes encoding for *FLT3LG* within the tumor are linked to NK cell presence. In melanoma patients, the presence of cDC1s and NK cells within the tumor correlate with increased survival and responsiveness to anti-PD1 treatment. This underscores the hypothesis that NK cells producing FLT3L are responsible for the abundance of cDC1s within the tumor resulting in improved patient survival (94).

**Figure 5:**
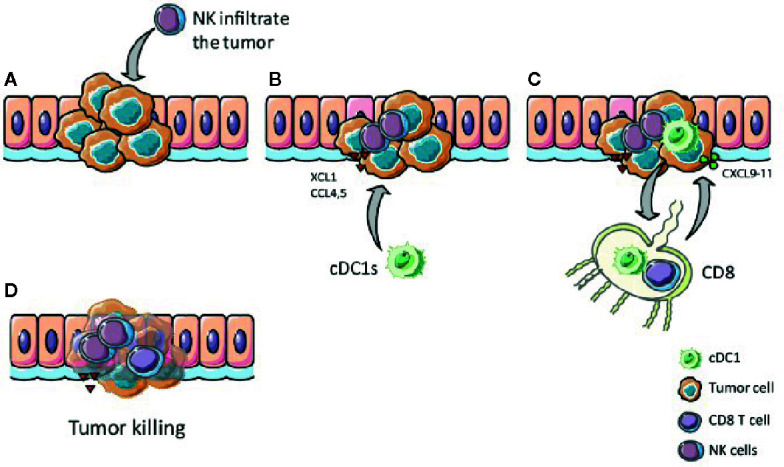
The XCR1 – XCL1 axis plays a role in tumor clearance. **(A)** NK and T cells, which infiltrate the tumor, produce XCL1 upon stimulation. **(B)** XCL1 attracts XCR1 expressing cDC1s to the tumor **(C)** cDC1s internalize, process, and cross-present tumor antigens to CD8+ T cells in the lymph nodes, thereby activate CD8+ T cells, which migrate to the tumor attracted by CXCL9-11 secreted by local cDC1s. **(D)** Activated CD8+ T cells kill tumor cells antigen-specifically

Taken together, chemokines such as XCL1, CCL4, and CCL5 secreted by NK cells and activated T cells within the TME recruit cDC1s into the tumor where they efficiently process antigens and then migrate to lymph nodes while they undergo maturation. In the draining lymph nodes, cDC1s prime naïve CD8^+^ T cells *via* cross-presentation. The cytotoxic T cells expand and are attracted to the tumor site, guided by local cDC1s secreting CXCL9 and CXCL10 (**Figure 5**). Thus, NK cells, cDC1s and their interaction are crucial for anti-tumor activity and, therefore, are promising targets to improve cancer immunotherapy outcome (6, 14, 37, 122).

## Manipulation of NK-cDC1 Cross Talk

### Targeting cDC1s and XCR1-XCL1 Axis

**Figure 6:**
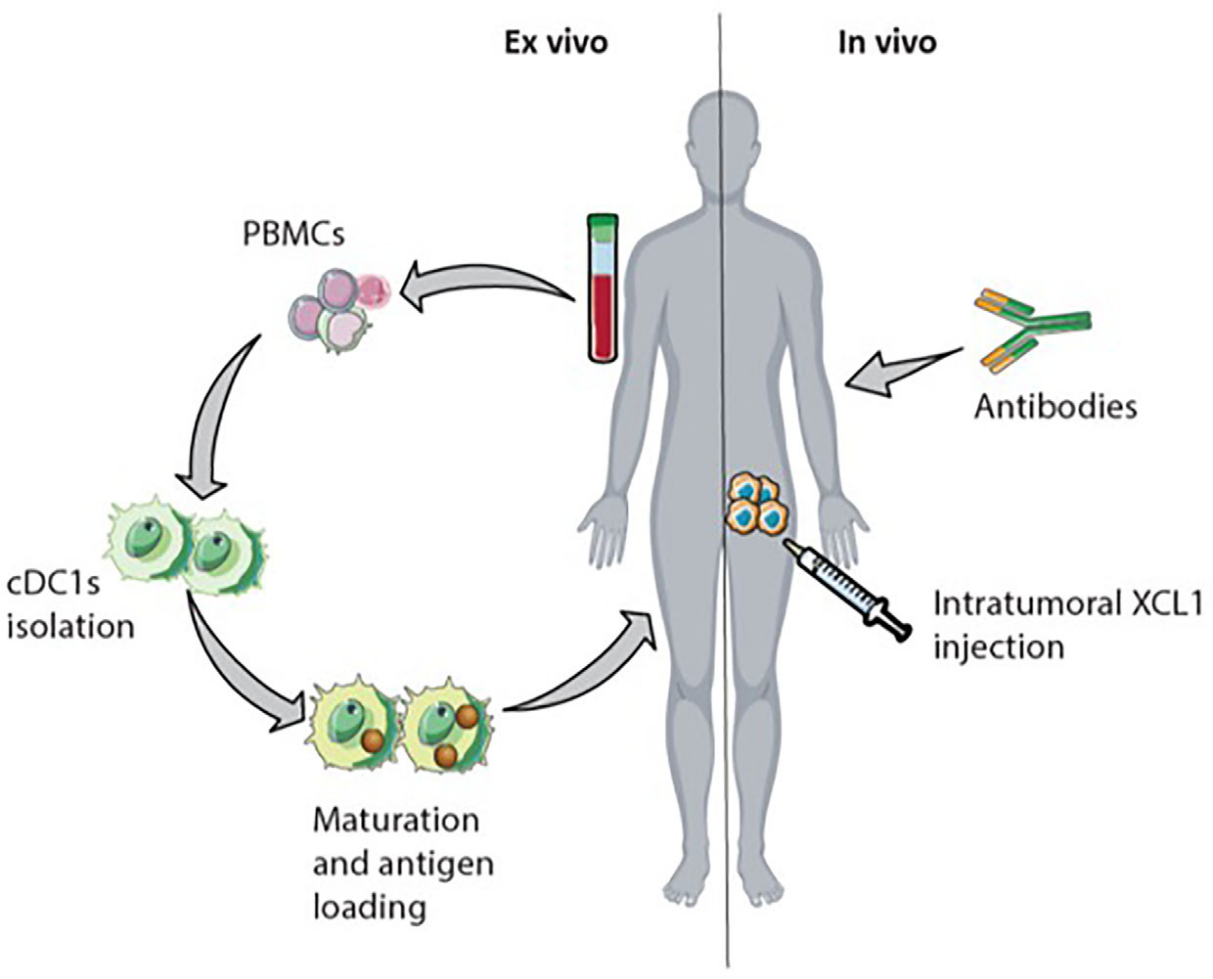
Possible immunotherapeutic approach targeting natural killer (NK) cells and cDC1s. Interfering with the XCR1-XCL1 axis in the tumor could increase antigen-specific CD8+ T cell infiltration. The intranodal reinfusion of cDC1s from patients after isolation them from PBMCs, maturation, and antigen-loading could be an *ex vivo* approach. *In vivo*, intratumoral XCL1 injection could recruit cDC1s into the tumor. Injected or attracted cross-presenting cDC1s in the tumor enhance CD8+ T cells activation. Monoclonal antibodies initiate antibody-dependent cellular cytotoxicity (ADCC) by NK cells leading to increased cross-presentation of tumor-cell derived antigens by cDC1s.

The presence and interaction of cDC1s and NK cells within the TME are associated with activation and increased cytotoxicity of NK cells, cDC1s infiltration and maturation, and better prognosis for cancer patients (14, 93, 95). Hence, interfering with the XCR1-XCL1 axis could be an approach to increasecDC1s numbers and thereby also increase numbers of T cells and NK cells within the tumor (**Figure 6**). Activated intratumoral CD8^+^ T cells and NK cells produce XCL1, attracting XCR1 expressing cDC1s. The intratumoral injection of XCL1 can increase the migration of cDC1s towards the TME (91). In mice, administration of XCL1 linked with antigen (e.g., OVA) targeting cDC1s results in an antigen-specific T cell response and reduced tumor growth (123–125). Nonetheless, there is skepticism about using XCL1 to attract cDC1s because of its unstable structure and relatively weak chemotactic activity (126, 127). For this reason, Kazuhiko *et al*. engineered a stabilized, more potent agonist form of murine XCL1. Upon injection in mice, this potent version of XCL1 showed increased recruitment of XCR1^+^ DCs, compared to wild-type XCL1 (126).

Besides targeting XCL1 to increase CD8^+^ T cell and NK cell numbers within the TME, direct administration of cDC1s into the TME could also be an approach. In mice, the application of cDC1s loaded with tumor-cell derived antigens enhanced tumor T cell infiltration and reduced tumor cell growth (128). DC therapy in the clinic shifts the application of monocyte-derived DC towards the application of blood-derived cDC2s and the combination of cDC2s and pDCs (129). Even though cDC1s are associated with improved survival in different types of cancer, the application of cDC1s is not yet assessed due to their low frequency in human blood (14, 93, 104, 129). Proposed alternatives to overcome the low abundance of cDC1s are the *ex vivo* generation of cDC1s from progenitor cells or expanding cDC1 progenitors *in vivo* (94, 130, 131). *In vitro* stimulation of human hematopoietic progenitors with Notch signaling and FLT3L induces cDC differentiation and yields phenotypical cDC1s with cross-presenting abilities (132). In mice, injection of FLT3L and intratumoral TLR3 stimulus poly IC, leads to expansion of cDC1 progenitor cells in the bone marrow and promotes cDC1s accumulation within the TME (131).

### Monoclonal Antibody Treatment

Checkpoint blockade therapies targeting CTLA-4 and PD-1/PD-L1 are successfully used as cancer immunotherapy (133). PD-1 and CTLA-4 expression on NK cells is upregulated in several types of cancer and is associated with reduced cytotoxicity and cytokine secretion (66, 82, 134, 135). Especially in low MHC-I expressing tumors, the effect of mAbs blocking PD-1/PDL-1 might partly be facilitated by NK cells (136). Antibodies targeting PD-1 or CD40 stimulate IFN-γ secretion, which drives IL-12 production by cDC1s that licenses cytotoxic T cell responses in both mice and cancer patients (137). Mice lacking cDC1s display no specific T cell response upon mAbs targeting of PD-1, indicating that the effectiveness of anti-PD1 mAbs depends on the presence of cDC1s within the tumor (94, 131, 138). Moreover, the expression of PD-L1 on DC seems to influence the efficacy of anti-PD-L1 mAb treatment as mice lacking PD-L1 expression on DCs show no response to anti-PD-L1 mAb treatment (100). Based on pre-clinical data, it is proposed that therapies recruiting and activating NK cells within the TME could increase cDC1s within the tumor, benefitting responsiveness to checkpoint inhibitors (94). In line with that, enhanced NK cell activity in mice resulted in increased anti-PD-1 and anti-CTLA4 responses and a better control of tumor growth (139). In melanoma patients, anti-PD-1 treatment response correlates with the presence of cDC1s and NK cells within the TME (94).

Other mAbs target tumor-associated antigens like Her2/neu which are overexpressed on malignant cells compared to healthy cells (140). Monoclonal Ab administration induces NK cell-mediated ADCC (47). NK cells activated *via* ADCC release soluble factors like cytotoxic granules and cytokines (IFN-γ and TNF-α). The uptake of cytotoxic granules initiates tumor cell apoptosis causing the release of tumor-cell derived antigens, which can be taken up and presented by DCs (47, 141). Simultaneously, IFN-γ and TNF-α secreted by NK cells activate cDC1s to cross-present antigens leading to an antigen-specific CD8^+^ T cell activation (47, 142). Moreover, mAb-activated NK cells can enhance NK cell-cDC1 cross-talk by activating cDC1s to secrete IL-12 which amplifies NK cell activation (141).

In some tumor types, inhibitory receptors such as KIRs and NKG2A are upregulated on NK cells (3, 143, 144). Therefore, mAb against these inhibitory receptors could be targets for blocking immune inhibition and thereby increase the cytotoxic potential of NK cells and anti-tumor immunity (145). mAbs targeting KIRs, NKGA2, and other inhibitory receptors are currently investigated in clinical trials and are until now considered safe with limited side effects (3, 145).

## Conclusion

Both cDC1s and NK cells are important for a proper anti-tumor immune response. cDC1s due to their specialization in cross-presenting tumor antigens and initiating an antigen-specific T cell response. NK cells because of cytotoxic and immunomodulatory functions. Even though both cell types can be affected by immunosuppressive factors in the TME, the NK-cDC1 cross-talk within this microenvironment establishes the cooperative nature of protective immunity against tumors. cDC1s, as producer of IL-12, CXCR9, CXCR10, and CCL5, attract NK cells to the TME, initiate cytokine production and boost NK cell cytotoxicity. At the same time, NK cells enhance cDC1 accumulation in the TME *via* XCL1 and CCL4, CCL5 secretion, and favor differentiation of cDC precursors to cDC1s. NK cells further stimulate DC maturation *via* cytokine secretion and CD40 ligation. Moreover, NK cell-mediated lysis of target cell releases cell debris including tumor-antigens, processed by cDC1s and cross-presented to CD8^+^ T cells. Thus, NK cell-cDC1 cross-talk is a promising target for immunotherapy to improve the clinical outcome of cancer patients.

## Author Contributions

JB wrote the manuscript and designed the figures. TZ and RS helped with the literature and wrote the manuscript. GS, IV, and GF-G designed and reviewed the manuscript. All authors contributed to the article and approved the submitted version.

## Funding

NHealth Holland/SGF grant DC4Balance (LSHM18056-SGF) and EU grant PROCROP (grant nr. 635122).

## Acknowledgement

Images were self-made using the server medical art. 

## Conflict of Interest

The authors declare that the research was conducted in the absence of any commercial or financial relationships that could be construed as a potential conflict of interest.
